# Clusterin protects neurons against intracellular proteotoxicity

**DOI:** 10.1186/s40478-017-0481-1

**Published:** 2017-11-07

**Authors:** Jenna M. Gregory, Daniel R. Whiten, Rebecca A. Brown, Teresa P. Barros, Janet R. Kumita, Justin J. Yerbury, Sandeep Satapathy, Karina McDade, Colin Smith, Leila M. Luheshi, Christopher M. Dobson, Mark R. Wilson

**Affiliations:** 1 Centre for Clinical Brain Sciences, University of Edinburgh, Chancellor’s Building, Edinburgh, EH16 4SB UK; 2 Euan MacDonald Centre for MND Research, 49 Little France Crescent-Chancellor, Edinburgh, EH16 4SB UK; 3 Department of Chemistry, University of Cambridge, Lensfield Road, Cambridge, CB2 1EW UK; 4 Illawarra Health and Medical Research Institute, University of Wollongong, Wollongong, NSW 2522 Australia

**Keywords:** TDP-43, Cytoplasmic inclusions, Proteotoxicity, Chaperone translocation

## Abstract

It is now widely accepted in the field that the normally secreted chaperone clusterin is redirected to the cytosol during endoplasmic reticulum (ER) stress, although the physiological function(s) of this physical relocation remain unknown. We have examined in this study whether or not increased expression of clusterin is able to protect neuronal cells against intracellular protein aggregation and cytotoxicity, characteristics that are strongly implicated in a range of neurodegenerative diseases. We used the amyotrophic lateral sclerosis-associated protein TDP-43 as a primary model to investigate the effects of clusterin on protein aggregation and neurotoxicity in complementary in vitro, neuronal cell and *Drosophila* systems. We have shown that clusterin directly interacts with TDP-43 in vitro and potently inhibits its aggregation, and observed that in ER stressed neuronal cells, clusterin co-localized with TDP-43 and specifically reduced the numbers of cytoplasmic inclusions. We further showed that the expression of TDP-43 in transgenic *Drosophila* neurons induced ER stress and that co-expression of clusterin resulted in a dramatic clearance of mislocalized TDP-43 from motor neuron axons, partially rescued locomotor activity and significantly extended lifespan. We also showed that in *Drosophila* photoreceptor cells, clusterin co-expression gave ER stress-dependent protection against proteotoxicity arising from both Huntingtin-Q128 and mutant (R406W) human tau. We therefore conclude that increased expression of clusterin can provide an important defense against intracellular proteotoxicity under conditions that mimic specific features of neurodegenerative disease.

**Electronic supplementary material:**

The online version of this article (10.1186/s40478-017-0481-1) contains supplementary material, which is available to authorized users.

## Introduction

Protein misfolding, aggregation and deposition are unifying features of a wide range of neurodegenerative diseases [1]. The ability of neurons to manage the burden of misfolded proteins and to resist their accumulation into insoluble protein deposits depends critically on the functioning of molecular chaperones. Previous studies have demonstrated that elevation of chaperone levels can protect against neurotoxicity resulting from the effects of pathological protein misfolding in cell culture and in transgenic animal models [2, 3]. Most chaperones are localised within intracellular compartments, although some are secreted into the extracellular environment. Prominent amongst such extracellular chaperones is clusterin (CLU), which is present in both plasma and cerebrospinal fluid (CSF). CLU is a cytoprotective chaperone whose expression level is increased in response to a diverse range of stresses including heat, pro-apoptotic insults, oxidative stress, ionising radiation, and proteotoxicity [4–6]. It has been linked to a correspondingly diverse group of clinical disorders associated with protein misfolding including Alzheimer’s disease (AD) [7], amyloidotic cardiomyopathy [8] and familial amyloidotic polyneuropathy [9]. CLU binds promiscuously to a wide range of misfolded client proteins and either sequesters them into stable, soluble complexes (in the case of proteins forming amorphous aggregates) or inhibits the formation and accumulation of toxic amyloid assemblies [10, 11]. Clusterin is a particularly potent chaperone and can inhibit protein aggregation at molar ratios of chaperone:client protein that are significantly lower than those needed by other chaperones [12, 13]. Extracellular CLU-client protein complexes are susceptible to endocytic clearance by monocytes and hepatocytes, and this action is thought to form a central plank in the operation of a co-ordinated system to maintain extracellular protein homeostasis (proteostasis) [14].

Whilst CLU is predominantly a secreted protein, multiple independent studies have now clearly established that during conditions of ER stress, CLU secretion is inhibited, and full-length CLU exits the secretory system to enter the cytosol. Firstly, the translocation of mature CLU from the ER/Golgi into the cytosol of human astrocytoma U251 cells in response to ER stress was shown by two complementary biochemical and immunofluorescence approaches [15]. Subsequently it was demonstrated that the ER-resident chaperone BiP (GRP78) facilitates the translocation of CLU to the cytosol during ER stress in human prostate cancer LNCaP cells [16]. In addition, it was recently shown that during ER stress in PC3 and LNCaP cells, cytoplasmic CLU interacts with LC3 to promote autophagy [17]. While the mechanism by which CLU translocates from the ER lumen to the cytosol in response to stress is yet to be elucidated, it appears that the release may form a part of a larger strategy in which many different chaperones are released from the ER to the cytosol to defend the cell against intracellular stresses. This has been suggested by the demonstration that, in response to cytosolic foreign DNA or inhibition of the proteasome, BiP is released to the cytosol [18]. Other earlier studies have also suggested that CLU might be involved in stress responses within the cell. For example, CLU accumulates in cells when either lysosomal degradation or the proteasome are specifically inhibited [19], and CLU is itself ubiquitinated during ER stress (a process that can only occur in the cytosol) [15]. Furthermore, other studies have suggested that CLU promotes ubiquitination and proteosomal degradation of the cytosolic proteins, COMMD1 and IkB [20] and the lysosome-mediated degradation of the cell-surface copper transport proteins, ATP7A and ATP7B [21, 22]. Collectively, the previous literature points to a potential involvement of CLU, especially during ER stress, in facilitating the degradation of specific cell proteins via both of the primary cell degradative pathways, the proteasome and autophagy.

The pathologies of many neurodegenerative diseases are known to involve both ER stress and cytotoxic protein aggregation [23]. Consequently, we examined whether increased expression of the unusually potent chaperone CLU could provide protection against intraneuronal protein aggregation and proteotoxicity, especially under conditions of ER stress. As a primary model, we studied the aggregation of the 43 kDa transactive response DNA-binding protein (TDP-43), which is implicated in the pathology of amyotrophic lateral sclerosis (ALS), where it translocates from its normal location in the nucleus and forms insoluble inclusions in the cytoplasm. We investigated the ability of CLU to influence the aggregation and clearance of TDP-43 in vitro, in neuronal cells and in transgenic *Drosophila melanogaster*. The results indicate that CLU can interact with TDP-43 and potently inhibit its aggregation, and that in a neuronal cell model undergoing ER stress, CLU reduces the numbers of cytoplasmic TDP-43 inclusions. We further show in transgenic *Drosophila* that co-expression of CLU reduces cytoplasmic mislocalization of TDP-43 in motor neurons, protects against loss of locomotor activity and significantly extends lifespan. Lastly, we showed that in *Drosophila* photoreceptor cells, CLU gave ER stress-dependent protection against proteotoxicity arising from both Huntingtin-Q128 and mutant (R406W) human tau.

## Materials and methods

### Cell culture and in vitro methods

#### Transfection, immunocytochemistry and confocal imaging of mammalian cells

SH-SY5Y, N2a and U251 cells were grown in DMEM/F12 (Life Technologies) supplemented with 10% (*v*/v) foetal calf serum (Bovogen) either on glass coverslips in 12 well microplates or 8 well glass bottom μ-Slides (Ibidi). When cells were 60-80% confluent they were transfected as indicated with either pCAG-EGFP/RFP **(**encoding the wild-type TDP-43-tdTomato fusion protein (Addgene plasmid 28,205) using X-tremeGENE HP (Roche), pRc/CMV-HT7 (encoding human CLU; [24]), pEGFP-N1-TDP-CTF (encoding a ~ 20 kDa C-terminal fragment (residues 216-414) of human TDP-43 fused to enhanced green fluorescent protein (EGFP); Addgene plasmid #28197) or pCMV6-AC-(M337 V)TDP-43-tGFP (Origene; M337 V mutation introduced into the wild type human TDP-43 sequence, fused at the C-terminus to turboGFP) using Lipofectamine 2000 (Life Technologies) according to the manufacturer’s instructions. 48 h after transfection, cells were treated (or left untreated) for 10 h with 2.5 μM A23187, 2.75 μM Tg and/or 10 μM MG132 (all from Sigma). The cells were immunostained for CLU as follows. Cells were first chemically fixed by incubation for 15 min on ice in 4% (*w*/*v*) paraformaldehyde in phosphate buffered saline (PBS; 135 mM NaCl, 10 mM Na_2_PO_4_, 2.7 mM KCl, 1.75 mM KH_2_PO_4_, pH 7.4), then permeabilized by a 20 min incubation on ice in 0.5% (*v*/v) TX-100 in PBS. Mouse hybridoma culture supernatants containing IgG1 G7 (anti-human CLU) or DNP9 (anti-2,4-dinitrophenyl) monoclonal antibodies [11] (both diluted 1:2 in 1% *w*/*v* bovine serum albumin (BSA) in PBS) were used as primary antibodies. These were detected using goat anti-mouse Ig conjugated with Alexa Fluor-488 or Alexa Fluor-555 IgG (ab150113 and ab150114, Abcam) (2 μg/ml). The nuclei were then stained with RedDot2 (Biotium) according to the manufacturer’s instructions. The cells were washed with PBS after each staining step. Cells grown on coverslips were mounted on a glass slide using Citifluor™ CFPVOH and AF100 anti-fadent (ProSciTech). Imaging was performed on a Leica TCS SP5 II confocal microscope using Leica Application Suite Advanced Fluorescence version 2.6.1-7314. Sequential excitation was performed using 488 nm, 561 nm and 633 nm lasers and fluorescence emissions collected at 500-540 nm (for the 488 nm laser), 570-620 nm (for the 561 nm laser) and 650-750 nm (for the 633 nm laser). In co-localization analyses, to determine the Manders’ overlap coefficient, images were first background subtracted using ImageJ. Regions of interest were then drawn around the cells to exclude pixels lacking intensity in both fluorescence channels (zero - zero pixels) and the extent of co-localization was quantified using the Coloc 2 function in ImageJ with Costes thresholding.

#### Immunoprecipitation of CLU from N2a cell lysates

N2a cells were transfected to express TDP-43^M337V^-tGFP (Origene) and human CLU using Lipofectamine 2000 transfection reagent according to the manufacturer’s instructions (Life Technologies). Some cells were transfected to express only TDP-43^M337V^-tGFP or CLU. Cells (200,000 in all cases) were harvested with trypsin/EDTA and washed twice with PBS (300 x *g*, 5 min) before being lysed on ice for 5 min in 150 μl PBS containing 1% (*v*/v) TX-100 and Complete Protease Inhibitor Cocktail (Roche). Any insoluble material was pelleted (21,000 x g, 15 min, 4 °C) and the cleared lysate (130 μl) was gently mixed overnight at 4 °C with Sepharose beads (~ 20 μl packed volume) coupled with mouse monoclonal G7 anti-CLU antibody. In some cases purified human CLU or BSA (100 nM) was added directly to the lysate immediately prior to the addition of the Sepharose beads. The beads were then washed 4 times in PBS (10 min, 2000 x *g*, 4 °C) before the bound proteins were eluted from the beads by boiling for 5 min in 25 μl SDS sample buffer. The beads were removed by centrifugation (2000 x *g*, 10 min) and the eluted proteins (~ 20 μl) analysed by Western blotting for the presence of TDP-43 as described below.

#### Western blotting

Proteins electrophoresed through a 10% SDS-polyacrylamide gel were transferred onto a nitrocellulose membrane (Sartorius) at 4 °C overnight (20 V) using a Western Transfer Unit (BioRad). The membrane was then blocked for 1 h at room temperature (RT) with blocking buffer (5% (*w*/*v*) skim milk in PBS containing 0.1% (*v*/v) TX-100). The membrane was then incubated with TARDBP monoclonal antibody (clone 2E2-D3, Abnova; 1:500) followed by an HRP-conjugated goat-anti-mouse IgG antibody (DAKO; 1:2000). Each antibody was diluted into blocking buffer, and each incubation was followed by washing the membrane 3X with PBS containing 0.1% (*v*/v) TX-100, followed by 3X washes with PBS. Bound antibodies were detected using Supersignal West Pico Chemiluminescence Substrate (Thermo Fisher Scientific), according to the manufacturer’s instructions. Bands were detected using X-ray film (Amersham Hyperfilm; GE Life Sciences).

#### Measurement of TDP-43 inclusions in N2a cells

This was done essentially using the method described in [25], which uses flow cytometric analysis to determine the transfection efficiency, and in cell lysates enumerate (i) fluorescent inclusions formed by the aggregation of fluorescent fusion proteins, and (ii) concurrently, the number of cell nuclei. This allows calculation of the number of fluorescent protein inclusions per 100 transfected cell nuclei. N2a cells grown in 24 well plates were transfected to express TDP-43^M337V^-GFP and cultured for 10 h with or without thapsigargin (2.75 μM), A23187 (2.5 μM), MG132 (10 μM), chloroquine (50 μM), bafilomycin A1 (100 nM), U0126 (60 nM), rapamycin (1.5 μM), or combinations of the preceding. Cells were then harvested using 0.5% trypsin/EDTA, washed and resuspended in 500 μl of PBS. To estimate transfection efficiency, a 150 μl aliquot of the cell suspension was analysed, together with an aliquot of untransfected N2a cells, by flow cytometry using an LSRFortessa X-20 (BD Bioscience). GFP fluorescence was measured using 488 nm excitation and 525/50 nm emission. The remaining cells were lysed in 0.5% (*v*/v) Triton X-100 in PBS, containing RedDot2 (1:1000; Biotium) to stain nuclei and Complete protease inhibitor (Roche). The lysate was analyzed by flow cytometry measuring forward and side scatter, and the fluorescence of GFP (acquired as above) and RedDot2 (640 nm excitation, 670/30 nm emission); data was analyzed as described above.

#### In vitro aggregation assay for TDP-43 peptide

CLU was purified from human plasma obtained from Wollongong Hospital (Wollongong, NSW, Australia) as previously described [11]. The synthetic peptide corresponding to residues 286-331 of TDP-43 (TDP-43^286-331^) was obtained from China Peptides and Thioflavin T (ThioT) from Sigma**.** TDP-43^286-331^ was dissolved in MilliQ water (adjusted with NaOH to pH 11.3) followed by the addition of a one tenth volume of buffer (1 M NaCl, 0.5 M HEPES, pH 7.4), to give a final concentration of approximately 448 μM TDP-43^286-331^. This solution was then diluted with an equal volume of 100 mM NaCl, 50 mM HEPES, pH 7.4, containing other additions (or not) to give a final concentration of 224 μM TDP-43^286-331^ with or without 2 μM SOD1 or 0.22, 0.022, 0.011, or 0.008 μM CLU. These solutions were incubated at 37 °C whilst shaking for 16 h in a 384 well microplate. ThioT (20 μM) was added to each well prior to incubation in a FLUOstar OPTIMA (BMG LABTECH) with an excitation filter of 440 +/− 10 nm and an emission filter of 490 +/− 10 nm. SOD1 was also incubated without the addition of TDP-43 and no significant change in fluorescence emission was detected over the time course (data not shown).

#### In vitro translation and aggregation assay for TDP-43-tGFP

cDNA encoding a human TDP-43-tGFP construct was cloned into pT7CFE1-CHis (Thermo Fisher Scientific). Both the cloning and the verification of insertion by sequencing were performed by GenScript. Full length human TDP-43-tGFP was expressed using the TnT® T7 Quick Coupled Transcription/Translation System (Promega) in a POLARstar Omega plate reader (BMG LABTECH) according to the manufacturer’s instructions (30 °C for 90 min). After the reaction was complete a final yield of 43 nM was estimated (based on manufacturer’s specifications) and the mixture was centrifuged to remove any aggregated material (16,600 x *g*, 10 min, 4 °C). The supernatant was incubated at either 4 °C or 37 °C for 5 h to induce aggregation with or without the addition of CLU or BSA (both at 43 nM). Following this incubation the samples were centrifuged again as above to pellet any aggregated protein. The supernatant containing any soluble TDP-43-tGFP was collected and 5 μl was diluted 1:1 with 2X SDS sample buffer supplemented with 1% β-mercaptoethanol and boiled for 5 min. The proteins were separated by SDS-PAGE and analysed by Western blot, probing for TDP-43, as described above.

### Transgenic *Drosophila* methods

#### Generation of transgenic *Drosophila*

*Drosophila* expressing HA-tagged human TDP-43 were created previously [26]. Following the same procedure, the human CLU construct was codon optimised for expression in *Drosophila* and synthesised by Genscript, then cloned into the multiple cloning site of pUAST-AttB. Differences in expression of the constructs that could arise from their integration at different genomic loci were eliminated as the vectors contain sites for exploiting the PhiC31 system for site-specific integration of transgenes [27]. CLU expression was under the control of the *UAS-GAL4* system [28]. All injected constructs, including an empty pUAST plasmid for the control line, were incorporated at the same genomic locus (51D) on Chromosome II (Bestgene Inc.). All *Drosophila* lines were made isogenic by repeated backcrossing. Htt-Q128 and Htt-Q72-GFP flies were a gift of Hyung Don Ryoo (NYU). All other *Drosophila* stocks used were obtained from Bloomington Stock Centre.

#### Hemolymph extraction

*Drosophila* were decapitated and the bodies were placed with the thorax pointing downwards into a p200 pipette tip. Four decapitated *Drosophila* were placed sequentially into each tip and three tips were placed in to a 1.5 ml Eppendorf tube on ice. The Eppendorf tube was centrifuged for 15 min at 4000 x *g* in an Eppendorf desk-top microfuge at 4 °C. Approximately 1 μl of straw-coloured hemolymph was collected from each Eppendorf tube and was flash frozen in liquid nitrogen and stored at −80 °C. The total protein concentration of each hemolymph sample was determined by bicinchoninic acid microprotein assay and Western blot detection was carried out using 20 μg of hemolymph protein loaded per lane.

#### Deglycosylation of head lysate proteins

1-20 μg of protein (hemolymph or *Drosophila* head homogenate) was diluted in 20 μl H_2_O. To this solution 2 μl Nonidet P40 (NP40), 2 μl G7 buffer and 1 μl PNGaseF (all reagents from NEB deglycosylation kit) were added according to the manufacturer’s instructions. After gentle agitation for 6 h at RT, the samples were analysed by Western blotting under non-reducing conditions. These conditions are sufficient to remove virtually all sugars [29].

#### Western blot analysis of fly head homogenates

Five *Drosophila* per genotype were decapitated, homogenised in RIPA buffer: [50 mM Tris HCl at pH 8, 150 mM NaCl, 1% (*v*/v) TX-100, 0.5% (*w*/*v*) sodium deoxycholate, 0.1% (w/v) SDS and protease inhibitors (Roche), then centrifuged in an Eppendorf desk-top microfuge at 20000 x *g* for 30 min in order to pellet insoluble proteins. The pellets were dissolved in denaturing buffer (9 M urea, 1% SDS, 25 mM Tris, 1 mM EDTA) at 4 °C, sonicated at 42000 Hz for 30 s, and centrifuged as above for a further 30 min at 20000 x *g* to pellet any still insoluble proteins. The supernatant (urea-soluble proteins) was used as the insoluble fraction. Protein samples were separated on 4-12% Bis-Tris gels (Invitrogen) and transferred to PVDF membrane (Millipore). Blots were blocked with 5% (*w*/*v*) non-fat milk in 0.05% (*v*/v) TX-100/PBS, and then incubated with the following primary antibodies: rat anti-HA-biotin, High Affinity (3F10) antibody which reacts with the N-terminal HA-tag on the TDP-43 construct (Roche; 1:1000); rabbit polyclonal anti-TDP-43 antibody (Proteintech; 1:2500); mouse anti-CLU G7 and 41D [11] hybridoma culture supernatant (1:10); rabbit anti-phospho-eIF2alpha (Ser51) (Cell Signalling; 1:1000). Blots were washed 6 times for 10 min with gentle agitation at RT in 0.1% (*v*/v) TX-100/PBS and then incubated with anti-rat, anti-rabbit or anti-mouse secondary antibodies conjugated to HRP (DAKO; 1:5000). All antibodies were diluted in the blocking buffer specified above. Bands were detected using a SuperSignal® West Pico Substrate kit (Thermo Fisher Scientific).

#### Detection of UPR

A reporter construct, gift of Hyung Don Ryoo (NYU), was created so as to have an enhanced green fluorescent protein (EGFP) inserted after the IRE-1 splice site in XBP1, so that EGFP would only be in frame after XBP1 had been spliced by IRE-1; the splicing of XBP1 by IRE-1 only occurs when there is activation of the UPR, leading to EGFP expression [30]. We co-expressed this construct with each of TDP-43, Htt-Q128 and mutant (R406W) human tau, using a *gmr-GAL4* promoter and homogenized the heads of 10 adult *Drosophila* per experiment, 24 h following eclosion. These samples were then prepared for Western blot as above. The presence or absence of EGFP (and thus UPR activation) was detected by an EGFP specific antibody (mouse monoclonal anti-EGFP antibody, Abcam; 1:2000) and an anti-mouse Ig-HRP secondary antibody as above. In similar experiments, the Western blots were instead probed with rabbit anti-phospho-eIF2alpha (Ser51) antibody (Cell Signaling Technology; 1:1000) to detect phosphorylated eIF2α, an independent marker of the UPR [31].

#### *Drosophila* immunohistochemistry

Third-instar larval imaginal eye discs and adult eyes were dissected in PBS, fixed in 4% (*w*/*v*) paraformaldehyde (PFA) in 0.05% (*v*/v) TX-100 in PBS for 20 min at RT then permeabilised for 20 min at RT in 0.5% (v/v) TX-100/PBS, before blocking for 30 min in 5% (w/v) BSA in 0.05% TX-100/PBS. Subsequently, fixed and permeabilised discs were incubated overnight with rat anti-HA-biotin high affinity antibody (Clone 3F10, Roche) and mouse anti-CLU antibody (G7), as above. Samples were then incubated overnight in streptavidin Alexa Fluor 594 conjugate (Invitrogen; 1:10,000) and anti-mouse Alexa Fluor 488 conjugate (Invitrogen; 1:1000). All antibodies were diluted in the blocking buffer described above. Tissue was counterstained with TOTO-3 (Invitrogen; 1:10,000) diluted in 0.05% TX-100/PBS to detect nucleic acids.

#### Light and scanning electron microscopy of *Drosophila* eyes

*Light and fluorescence microscopy: Drosophila* expressing TDP-43 +/− CLU or Htt-Q72-GFP +/− CLU were crossed with *gmr-GAL4 Drosophila* (Bloomington Stock ID: 1104 & 8121) and maintained in a temperature and humidity controlled incubator at 25°C and 70% humidity. Images were taken of 1-day-old transgenic offspring using 7.5X objective and a Leica epifluorescence microscope; imaginal eye disks prepared as above were imaged using a Leica SP confocal microscope. *Scanning electron microscopy:* Samples were prepared by fixing whole adult *Drosophila* overnight in 2.5% glutaraldehyde in 0.1 M PBS (pH 7.4) at 4°C, then dehydrated with an ethanol series. Finally, samples were mounted on stumps and sputter coated using 20 nM Au/Pd in a Polaron E5000. SEM images were collected using a Philips XL30 microscope at 200× magnification.

#### Climbing assays

Motor function was assessed by a negative geotaxis assay. *Drosophila* were generated that expressed TDP-43 (with and without CLU), or CLU alone, in motor neurons from the day of hatching. Non-transgenic *Drosophila* were also tested as a control group. For each treatment group, three vials each containing ten *Drosophila* were analysed every second day. A climbing index score was calculated as described previously [32]. The average climbing index for the three replicate analyses was calculated for each time point and plotted against time since eclosion (*n* = 30).

#### Survival assays

*Drosophila* were generated at 18°C where Gal80 inhibits *GAL4* dependent transcription, thus preventing expression of transgenes in embryos and larvae. Adult *Drosophila* were moved from 18°C to 29°C (Gal80 is inactive at this temperature and so no longer inhibits expression of transgenes) within 24 h of eclosion; they were then transferred to fresh food and counted every 2-3 days. *Gal80; D42-GAL4* is activated by heat shock at 29°C and induces expression in motor neurons. Each treatment group was comprised of 90 non-virgin female *Drosophila* maintained in glass vials (10 per vial). Median survivals were calculated using Kaplan Meier survival statistics and differences between genotypes were analysed using a Mann-Whitney U test.

## Results

### ER stress induces CLU to co-localize with cytoplasmic TDP-43-GFP inclusions

Previous studies have shown that ER stress induces release of CLU to the cytosol [15–17]. We confirmed that, as expected, CLU was detected in the cytosol fraction prepared from N2a cells chemically treated to induce ER stress but not in the same fraction prepared from untreated cells (Additional file 1: Figure S1). Furthermore, analysis of the anterior horn cells (motor neurons) in human post-mortem spinal cord sections of three ALS patients and three age-and sex-matched control cases, revealed a striking difference in CLU staining, consistent with the release of CLU from the ER in ALS affected neurons (Additional file 1: Figure S2). In control cases CLU staining mirrored Nissl substance staining, consistent with its localization in the ER (secretory pathway). However, in the anterior horn cells of ALS cases, which exhibited prominent pathological cytoplasmic TDP-43 inclusions, CLU staining was increased and exhibited a more diffuse staining pattern. The pattern of CLU staining in these cells no longer mirrored the distribution of Nissl substance, consistent with a change in the subcellular localization of CLU (Additional file 1: Figure S2).

**Fig. 1:**
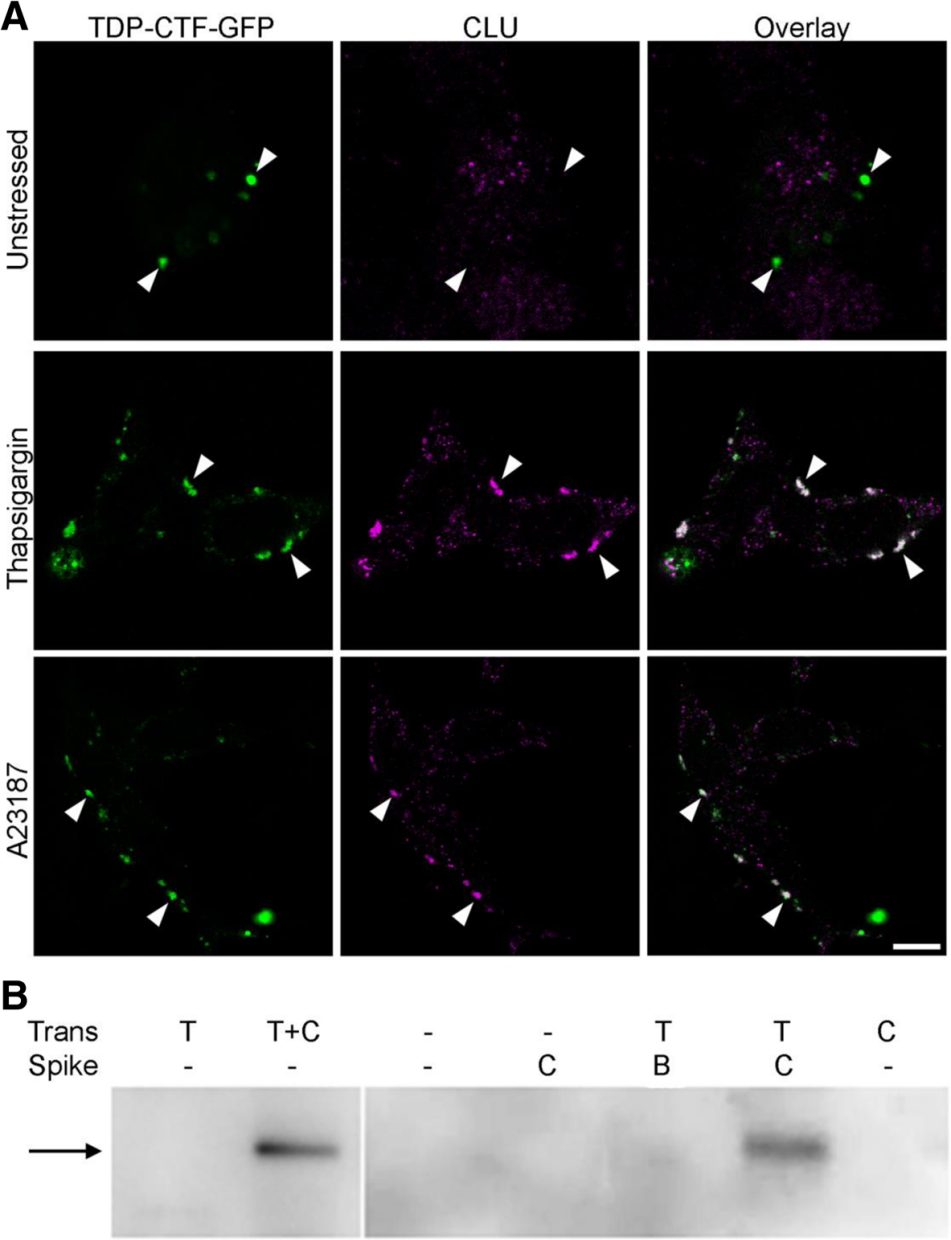
CLU co-localizes with cytoplasmic TDP-43 inclusions in ER stressed cells and co-precipitates with TDP-43-GFP. **a** N2a cells transfected to express TDP-43^CTF^-GFP and human CLU (hCLU) were treated as indicated, fixed/permeabilized, then immunostained for hCLU. White arrowheads indicate the positions of inclusions. Overlay panels (*right*): where there is no colocalization, inclusions appear green; colocalization of TDP^CTF^-GFP and CLU appears as white. Scale bar is 10 μm; images shown are representative of many. Manders overlap coefficients (TDP-43^CTF^-GFP and CLU), calculated using at least 20 cells for each treatment, were: untreated, 0.13 +/− 0.14; Tg, 0.62 +/− 0.07; A23187, 0.52 +/− 0.09 (mean +/− SD). See also Additional file 1: Figure S3. **b** TDP-43 immunoblot showing that TDP-43^M337V^-GFP co-precipitated with hCLU from lysates of co-transfected N2a cells and also when exogenous purified hCLU (but not BSA) was added (“spiked”) to lysate prepared from N2a cells transfected to express only TDP-43^M337V^. The black arrow indicates the expected size of TDP-43^M337V^-GFP. Key above image indicates which protein(s) cells were transfected to express (Trans), and the protein added to the lysate (Spike). The result shown is representative of two independent experiments

We next examined the effects of CLU over-expression in a cell model of protein mislocalization relevant to ALS. TDP-43 is a member of the hnRNP family of RNA binding proteins whose functions involve the regulation of RNA splicing and transcription. In almost all cases of sporadic and also in some familial cases of ALS, this predominantly nuclear protein becomes mislocalized in motor neurons to the cytoplasm, where it forms ubiquitinated and hyper-phosphorylated inclusions enriched in proteolytically generated C-terminal fragments of TDP-43 [33]. When N2a (or SH-SY5Y neuroblastoma) cells were transiently transfected to express an aggregation-prone C-terminal fragment of TDP-43 fused to GFP (TDP-43^CTF^-GFP), which forms aggregates with structural similarity to those in human post-mortem tissue and that is widely used as a tool to study TDP-43 aggregation [33], they developed cytoplasmic inclusions readily detected by confocal microscopy. Furthermore, consistent with ER stress inducing release of CLU to the cytosol, CLU was found co-localized with TDP-43^CTF^-GFP inclusions in ER stressed N2a cells, but not in unstressed N2a cells (Fig. 1a; Additional file 1: Figure S3). ER stress also induced co-localization of CLU with inclusions formed by a similar fusion protein (mutant M337 TDP-43 fused at the C-terminus with GFP; TDP-43^M337V^-GFP) in U251 human glioblastoma cells (Additional file 1: Figure S3). We also used co-immunoprecipitation analysis to show that soluble TDP-43^M337V^-GFP is physically associated with CLU in lysates prepared from co-transfected N2a cells, for both untreated cells (Fig. 1b) and cells treated to induce ER stress (not shown). Interestingly, we also used a monoclonal antibody specific for human CLU to show that exogenous human CLU added (“spiked”) to lysates of transfected N2a cells co-immunoprecipitated with TDP-43^M337V^-GFP. These results demonstrate that when CLU and TDP-43^M337V^-GFP are both present in cell lysates they can form complexes, regardless of whether or not the two proteins may have been in different cell compartments prior to lysis. Thus, while it is not possible to use a co-immunoprecipitation approach to verify the occurrence of CLU-TDP-43 complexes in situ inside cells, the results indicate that if the two proteins are present within the same compartment, such as suggested by the confocal images of cytoplasmic co-localization, they are capable of forming complexes.

### CLU potently inhibits TDP-43 aggregation in vitro

**Fig. 2:**
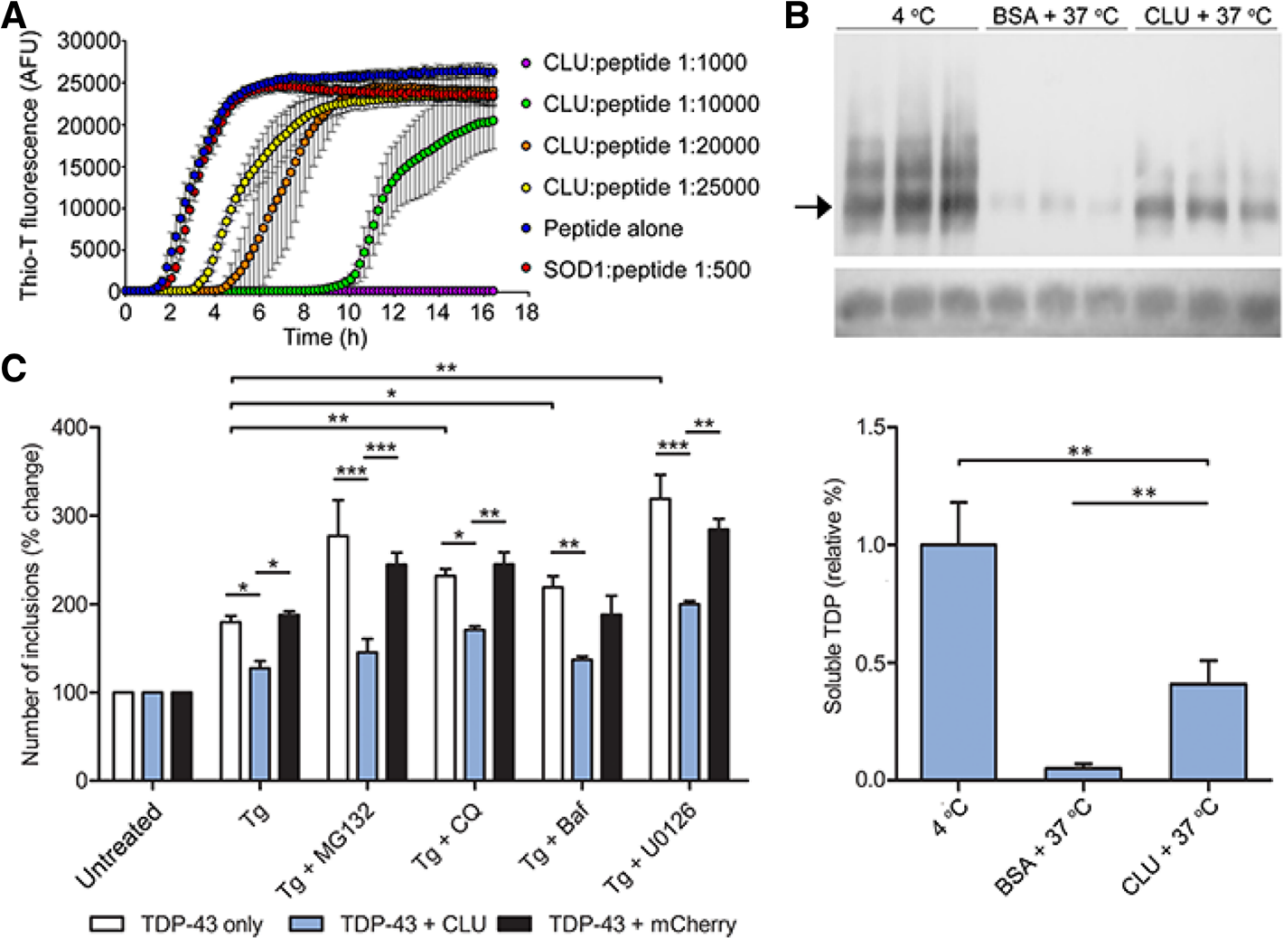
CLU inhibits TDP-43 aggregation and reduces the number of TDP-43-GFP inclusions in neuronal cells during ER stress. **a** TDP-43^286-331^ (peptide) was incubated at 37 °C with shaking, in the presence of either CLU or the non-chaperone control protein bovine superoxide dismutase (SOD1), at the molar ratios indicated in the key. Peptide aggregation was monitored by ThioT fluorescence. Data are means +/−SE (*n* = 4). **b** *Upper panel:* Image of Western blot detecting soluble TDP-43-tGFP after incubation at 4 °C, or 37 °C (which increases aggregation), with or without CLU or BSA (a non-chaperone control protein). Black arrow indicates position of monomeric TDP-43-tGFP, upper bands represent soluble oligomers. Loading control (colored dye from Promega transcription/translation kit) shown at bottom. *Lower panel:* Densitometric quantification of blot results (means + SD, *n* = 3). **c** Transfected N2a cells were treated as indicated for 10 h (key below plot), then lysed and TDP-43^M337V^-tGFP inclusions enumerated by flow cytometry; data plotted as % change relative to untreated cells (means + SEM, n = 4; untreated cells are defined as the 100% value). See also Additional file 1: Figure S5. For all panels, results shown are representative of two or more independent experiments. In (**b**) and (**c**), horizontal bars indicate pairwise comparisons, * *p* < 0.05, ** *p* < 0.01, *** *p* < 0.001 (1-way ANOVA with Newman-Keuls post-test (**b**), or 2-way ANOVA with a Bonferroni post-test (**c**))

Full length TDP-43 is so aggregation-prone that it is extremely difficult to express the full length protein in bacteria as a soluble recombinant product in significant quantities [34]. We therefore initially made use of a well-characterised 46-mer peptide corresponding to a region of the C-terminal domain of TDP-43 (TDP-43^286-331^), which is known to form fibrillar aggregates [35], to probe the ability of CLU to inhibit the in vitro aggregation of TDP-43. Under the conditions tested, following a 2 h lag phase, the TDP-43^286-331^ peptide rapidly formed ThioT positive aggregates. Under these same conditions, a CLU:peptide ratio of 1:1000 completely inhibited the appearance of ThioT positive species over a 16 h time course. Even at a 1:25,000 ratio, CLU retarded TDP-43^286-331^ fibril formation by about 2 h (Fig. 2a). This effect is specific to CLU, as even when used at a ratio of 1:500, a non-chaperone control protein (bovine superoxide dismutase 1, SOD1) had negligible effects on aggregation of the peptide (Fig. 2a). In order to confirm that CLU can also suppress the aggregation of full-length TDP-43, we adapted an in vitro protein expression system for full-length TDP-43-turboGFP (TDP-43-tGFP) described in [34]. Following in vitro expression, a significant amount of full-length TDP-43-tGFP remained soluble for 4 h at 4°C, but after a 4 h incubation at 37°C in the presence of 43 nM BSA (a non-chaperone control protein), very little TDP-43-tGFP remained soluble (Fig. 2b). When incubated for 4 h at 37 °C, the presence of 43 nM CLU resulted in an 8-fold higher level of TDP-43-tGFP remaining in solution (Fig. 2b), demonstrating that CLU is able to very significantly inhibit the aggregation of full-length TDP-43 in vitro.

### CLU expression specifically reduces the numbers of cytoplasmic TDP-43-GFP inclusions in ER stressed N2a cells

We used a recently developed flow cytometry method, which counts fluorescent particles in cell lysates and normalizes this count against the number of separately enumerated nuclei (FloIT; [25, 36, 37]) to quantify the numbers of TDP-43^M337V^-GFP inclusions in differently treated N2a cells. The results indicated that (i) in unstressed cells, overexpression of CLU or mCherry had no significant effect on the numbers of TDP-43^M337V^-GFP inclusions, (ii) the numbers of inclusions were increased in cells treated with either thapsigargin (Tg) alone (to induce ER stress), or with Tg and MG132 (the latter to also inhibit the proteasome), and (iii) overexpression of CLU, but not mCherry, reduced the numbers of TDP-43^M337V^-GFP inclusions in ER-stressed cells (Fig. 2c). CLU overexpression also specifically reduced the number of TDP-43^M337V^-GFP inclusions in N2a cells in which ER stress was induced by the addition of Ca^2+^ ionophore A23187 (Additional file 1: Figure S5A). The overexpression of CLU or mCherry had no significant effect on the numbers of inclusions in unstressed cells (Additional file 1: Figure S5B).

### Human CLU is glycosylated, cleaved and secreted when expressed in *Drosophila* neurons

**Fig. 3:**
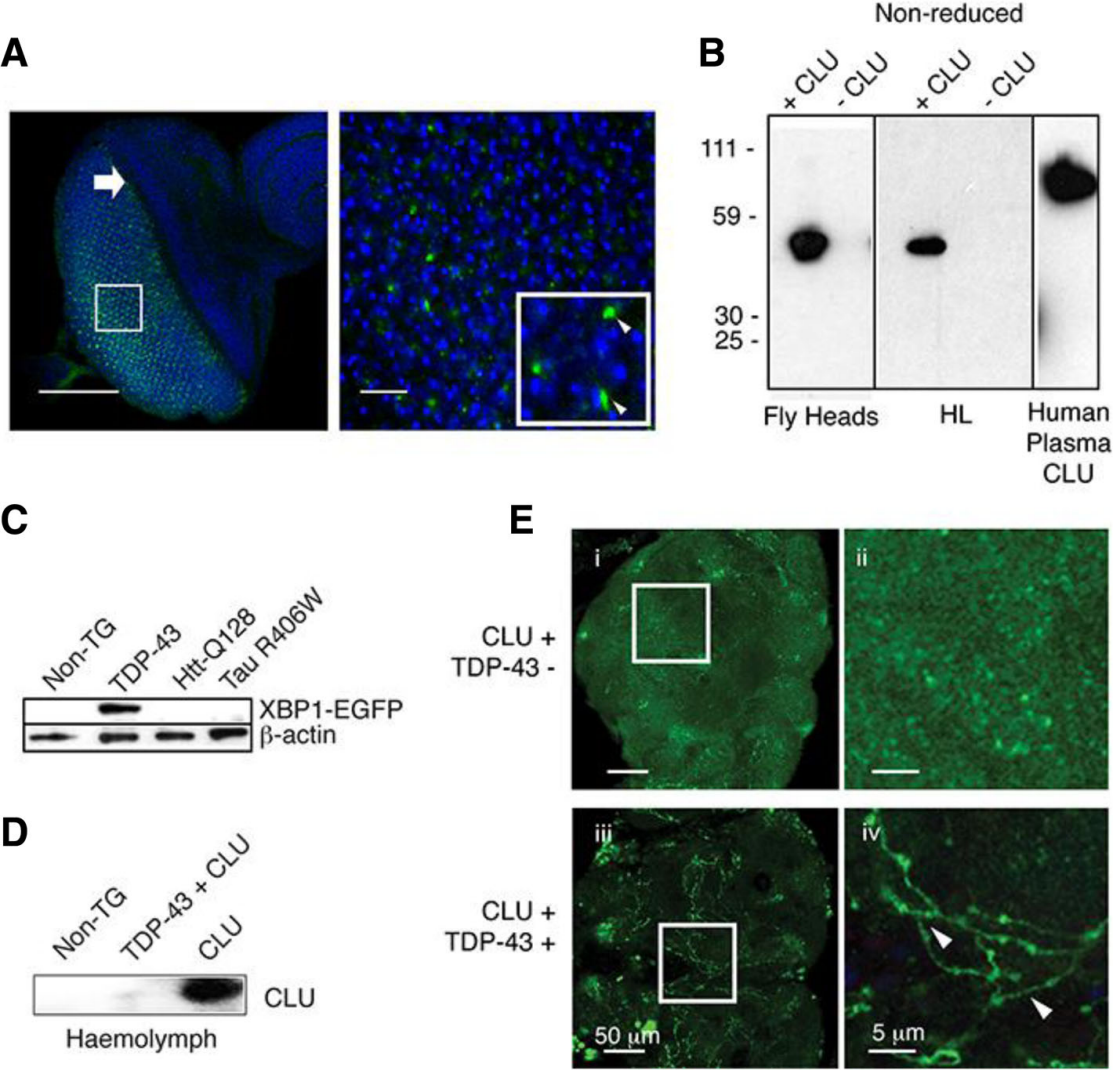
Expression of CLU and TDP-43 in *Drosophila*. **a** Confocal microscopy images of imaginal eye discs of third instar larvae expressing human CLU in the photoreceptor cells; CLU (*green*), TOTO-3 (*blue*, detects nuclei). *Left panel:* Large white arrow indicates the morphogenetic furrow (*scale bar* 100 μm); white box represents the area shown at higher magnification in the large panel to the right. *Right panel:* Punctate staining of CLU (*scale bar* 10 μm); the inset white box shows a region with still higher magnification (*arrowheads* indicate CLU). **b** Non-reducing Western blots of *Drosophila* head homogenates, hemolymph (HL), and the positive control (human plasma CLU); expression of CLU indicated above the lanes. **c** Western blot of whole *Drosophila* head lysates, prepared from non-transgenic (Non-TG) *Drosophila*, or *Drosophila* expressing TDP-43, Htt-Q128 or Tau R406W; detection of XBP1-EGFP indicates activation of the UPR (β-actin was used as a loading control). **d** Western blot detecting CLU in hemolymph taken from Non-TG *Drosophila*, and those expressing CLU +/− TDP-43; equal amounts of hemolymph were loaded in each lane under non-reducing conditions. See also Additional file 1: Figure S6. **e** Confocal microscopy images of the central lobe of 10-day-old, whole adult *Drosophila* brains expressing CLU +/− TDP-43 in the motor neurons. Images in (i) and (iii) encompass the entire central lobe; the central region in each image contains neuronal process and axons. Images in (ii) and (iv) represent optical zooms of the areas indicated by the white boxes in (i) and (iii), respectively. Scale bars in (i) and (iii) represent 50 μm, while those in (ii) and (iv) represent 5 μm. CLU (*green*), white arrowheads in (iv) indicate axons. Results shown are representative of two or more independent experiments

Given the ability of CLU to reduce the number of TDP-43 inclusions in cultured neuronal cells, we next developed a whole organism model in which the protective effects of CLU against cellular proteotoxicity could be further investigated. *Drosophila* has no identifiable homologue of clusterin. When expressed in photoreceptor neurons, human CLU was detected in juxtanuclear puncta distributed uniformly across the eye disc (Fig. 3a). This distribution is consistent with the protein being localised in the secretory pathway and analysis of the hemolymph (the circulatory fluid of *Drosophila*) confirmed that CLU is secreted from the neurons in which it is being expressed (Fig. 3b). Non-reducing SDS-PAGE and Western blot analysis of head lysates from *Drosophila* expressing CLU revealed that the protein was abundantly expressed and migrated as a single band with an apparent molecular mass of ~ 55 kD; it migrated at a lower molecular mass (~ 50 kD) when the sample was treated with PNGase to remove virtually all glycosylation (Additional file 1: Figure S6A). The molecular mass of CLU detected in the hemolymph and head lysates of *Drosophila* is, however, less than that of CLU purified from human plasma (~ 55 kD versus 75-80 kD), a difference attributable to the fact that insect cells glycosylate proteins to a lesser extent than mammalian cells [38]. Indeed, enzymatic deglycosylation of CLU expressed in *Drosophila* results in a smaller loss in apparent mass than for CLU from human plasma, and the deglycosylated forms of both proteins migrate at a very similar position when analyzed by SDS-PAGE (Additional file 1: Figure S6A). Differences in glycosylation do not, however, significantly affect the function of CLU, as indeed the removal of virtually all the attached carbohydrates does not inhibit its chaperone activity [29]. We also confirmed by SDS-PAGE that *Drosophila*-expressed CLU dissociates into α- and β-subunits when chemically reduced, as seen for CLU from human plasma (Additional file 1: Figure S6B). A faint CLU band detected at about 50 kDa may represent a minor glycoform of CLU, as glyco-variants of CLU are found in human plasma [39]. Taken together, these biochemical studies indicate that *Drosophila* neurons are able to synthesise and secrete a mature, post-translationally modified form of human CLU.

### TDP-43 expression induces ER stress and cellular retention of CLU in transgenic *Drosophila*

We first co-expressed both TDP-43 and a fluorescent reporter of the unfolded protein response (an EGFP-based reporter of the alternative splicing of XBP1 [30]) in photoreceptor neurons of the *Drosophila* eye. For comparison, we also examined the effects of expression both of exon 1 of the Htt gene, containing a 128 glutamine residue expansion (Htt-Q128; [40]), and also of mutant (R406W) human tau, which is associated with frontotemporal dementia with parkinsonism-17 (FTDP-17) [41]. Co-expression of the fluorescent UPR reporter with TDP-43, Htt-Q128 or R406W tau followed by analysis of EGFP levels by Western blotting, revealed that IRE1-dependent splicing of XBP1-EGFP was induced by expression of TDP-43 but not by the expression of either Htt-Q128 or R406W tau (Fig. 3c). The induction of ER stress by TDP-43 expression was also confirmed by Western blot analysis showing that the levels of phosphorylated eIF2α, an independent marker of the UPR [31], were much higher in homogenates of the heads of *Drosophila* expressing TDP-43 compared to those of control *Drosophila* (Additional file 1: Figure S6C). Western blotting was also used to confirm that CLU was secreted into the hemolymph when expressed alone in photoreceptor neurons, but that it was barely detectable in this fluid when co-expressed with TDP-43 in the same cells (Fig. 3d). We next expressed CLU in *Drosophila* motor neurons and examined its distribution in brains by confocal microscopy. Despite the limited resolution of confocal images obtained from the relatively thick adult *Drosophila* brain, it can be seen that, consistent with its secretion, CLU was diffusely distributed throughout the neuropil (Fig. 3e i & ii), and was not co-localized with neuronal cell bodies or axons. When co-expressed with TDP-43, however, CLU was found at high levels in the axons (Fig. 3e iii & iv).

### CLU restores nuclear localization of TDP-43 in adult *Drosophila* motor neurons

**Fig. 4:**
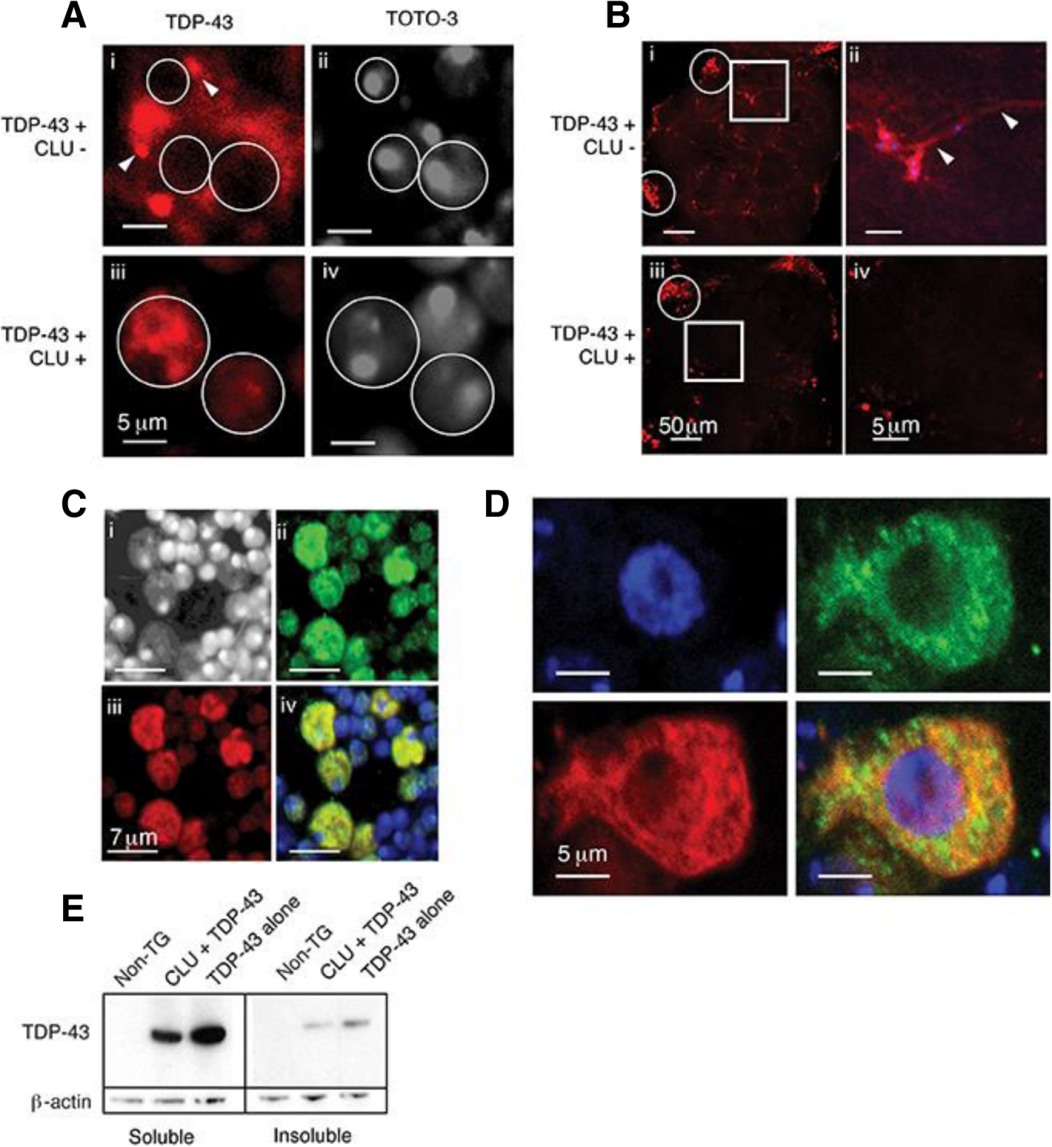
CLU expression reduces TDP-43 mislocalization in *Drosophila* neurons. Confocal microscopy images of: **a** Motor neuron cell bodies of 10-day-old adult *Drosophila* brains expressing TDP-43 alone (i & ii) or together with CLU (iii and iv). Images (i & ii) and (iii & iv) represent the same fields of view; TDP-43 (*red*; i & iii), TOTO-3 stained nuclei (*grey*; ii & iv). In (i), white arrowheads indicate paranuclear foci; in all panels white elipses indicate nuclei (*scale bars* 5 μm). **b** Central lobe of adult *Drosophila* brains expressing TDP-43 +/− CLU. Images in (i) and (iii) were collected using a 40X objective and encompass the entire central lobe (*scale bars* 50 μm); elipses show motor neuron cell bodies and the central region of each image contains neuronal process and axons. Images in (ii) and (iv) represent optical zooms of the areas indicated by the white boxes in (i) and (iii), respectively (*scale bars* 5 μm). TDP-43 (*red*), white arrowheads in (ii) indicate axons. **c** Motor neuron cell bodies of adult *Drosophila* brains co-expressing CLU (*green*) and TDP-43 (*red*); TOTO-3 (*white*; nucleoli stain intensely, the rest of the nucleus stains less brightly); (iv) overlay of all three images, yellow indicates co-localisation of CLU and TDP-43 (*scale bars* 7 μm). **d** Motor neuron cell bodies of third instar larval *Drosophila* brains co-expressing CLU (*green*) and TDP-43 (*red*), TOTO-3 (*blue*); (iv) overlay of all three images; yellow indicates co-localisation of CLU and TDP-43 (*scale bars* 5 μm). **e** Reducing Western blot of the soluble and insoluble fractions of 10-day-old adult *Drosophila* head homogenate comparing non-transgenic (non-TG) *Drosophila* and *Drosophila* expressing TDP-43 +/− CLU. β-actin was used as a loading control. Results shown are representative of several independent experiments

In the motor neurons of 10 day old adult *Drosophila* brains, when expressed alone, TDP-43 is frequently notably absent from its normal nuclear localization (elipses devoid of TDP-43; Fig. 4a i & ii) and is instead detected in foci localized within the cytoplasm of the motor neurons (white arrowheads; Fig. 4a i) as well as being diffusely distributed throughout the soma, axons and neuronal processes (white arrowheads; Fig. 4b i & ii). This observation is consistent with those made in yeast models in which high levels of expression of wild-type TDP-43, as achieved in the current *Drosophila* model, result in its mislocalization into the cytosol [42]. When CLU is co-expressed with TDP-43, however, the latter is no longer evident within the axons or neuronal processes and paranuclear foci of TDP-43 are no longer visible. Instead, TDP-43 is predominantly found within the nucleus (Fig. 4a iii & iv) where it strikingly co-localizes with CLU (Fig. 4c). At earlier stages of development, when the two proteins have been co-expressed for shorter periods of time, TDP-43 is largely absent from the nucleus and instead is found mainly in the cytoplasm where it extensively co-localises with CLU (Fig. 4d). Thus CLU co-expression results in the restoration in the adult *Drosophila* of a predominantly nuclear localization for TDP-43 and reduces the levels of both soluble and insoluble TDP-43 detected in head lysates (Fig. 4e)*.* This effect cannot be attributed to lowered expression of TDP-43 arising from transgene co-expression, as co-expression of TDP-43 with GFP (which has no chaperone activity) has no effect on TDP-43 levels (Additional file 1: Figure S7A).

### CLU protects *Drosophila* motor neurons from TDP-43-induced neurotoxicity

**Fig. 5:**
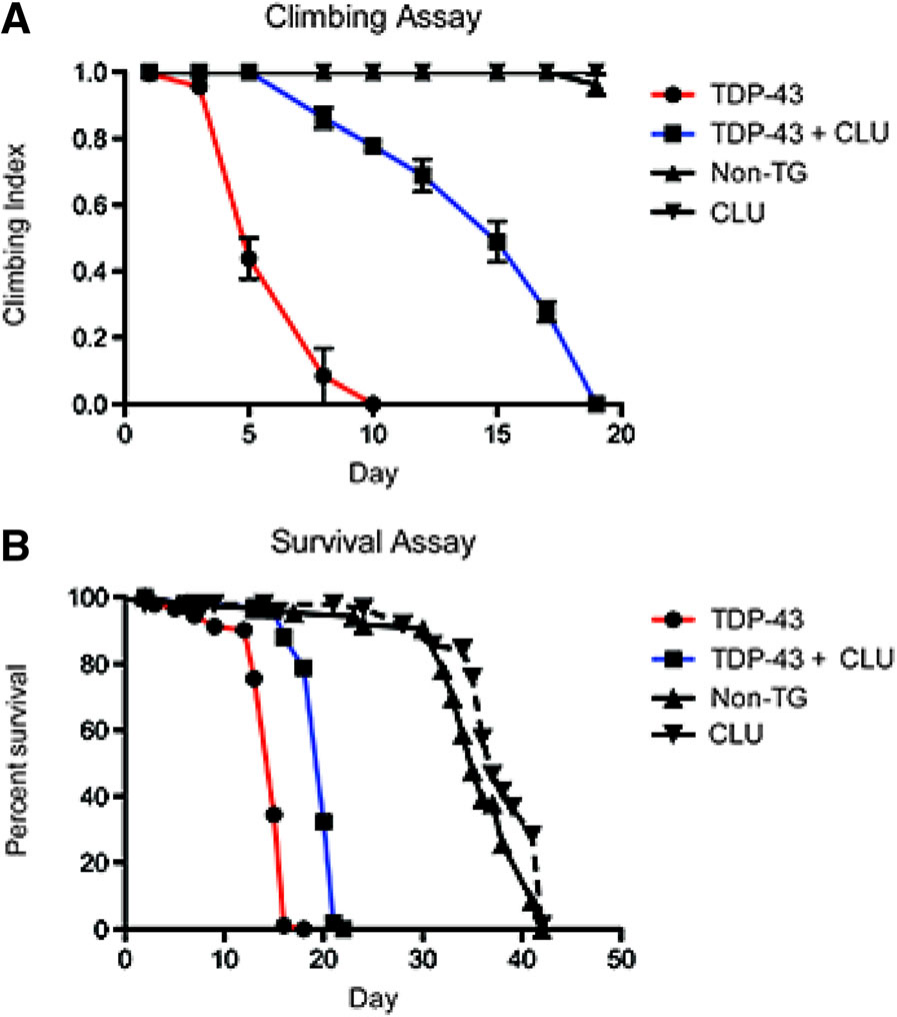
CLU expression reduces TDP-43 toxicity in *Drosophila* motor neurons. Time-dependent differences between *Drosophila* expressing TDP-43 +/− CLU in (**a**) motor function, measured by climbing assay, and (**b**) survival, compared to non-transgenic (Non-TG) *Drosophila*. In (**a**) and (**b**), values shown are means +/− SEM, and in each case the results shown are representative of three independent experiments. Parent lines used in crosses indicated in key. Differences in climbing index between genotypes were analysed by ANOVA (*n* = 30). Lifespans were analysed by Kaplan Meier statistics (*n* = 90). See also Additional file 1: Figure S7

We next investigated whether CLU expression could affect the neurodegenerative phenotypes resulting from intracellular expression of TDP-43 in *Drosophila* motor neurons. Strikingly, co-expression of CLU with TDP-43 in the motor neurons (i) significantly delays the onset of locomotor dysfunction, increasing the time taken for 50% of TDP-43 expressing *Drosophila* to become immobile from 5 days to 15 days (Fig. 5a), and (ii) increases the median survival time by 33% compared to *Drosophila* expressing TDP-43 alone (from 15 ± 0.39 days to 20 ± 0.53 days; *p* = 0.0006, *n* = 180; Fig. 5b). This rescue is specific to CLU expression, as co-expression of an unrelated protein (GFP; not expected to bind TDP-43) driven by the same *UAS-GAL4* system did not affect the median survival of TDP-43-expressing *Drosophila* (Additional file 1: Figure S7B). Furthermore, CLU expression alone did not cause a significant increase in the longevity of *Drosophila*. Non-transgenic *Drosophila* have a median survival of 36 days ±1.09 whereas CLU expressing *Drosophila* have a median survival of 37 days ±1.18 (*p* = 0.5056 ns, *n* = 180; Additional file 1: Figure S7C), indicating that the effects of CLU are on neurotoxicity induced by TDP-43 rather than representing a general effect on ageing.

### CLU-mediated protection against intracellular proteotoxicity is not restricted to TDP-43 and is dependent on ER stress

**Fig. 6:**
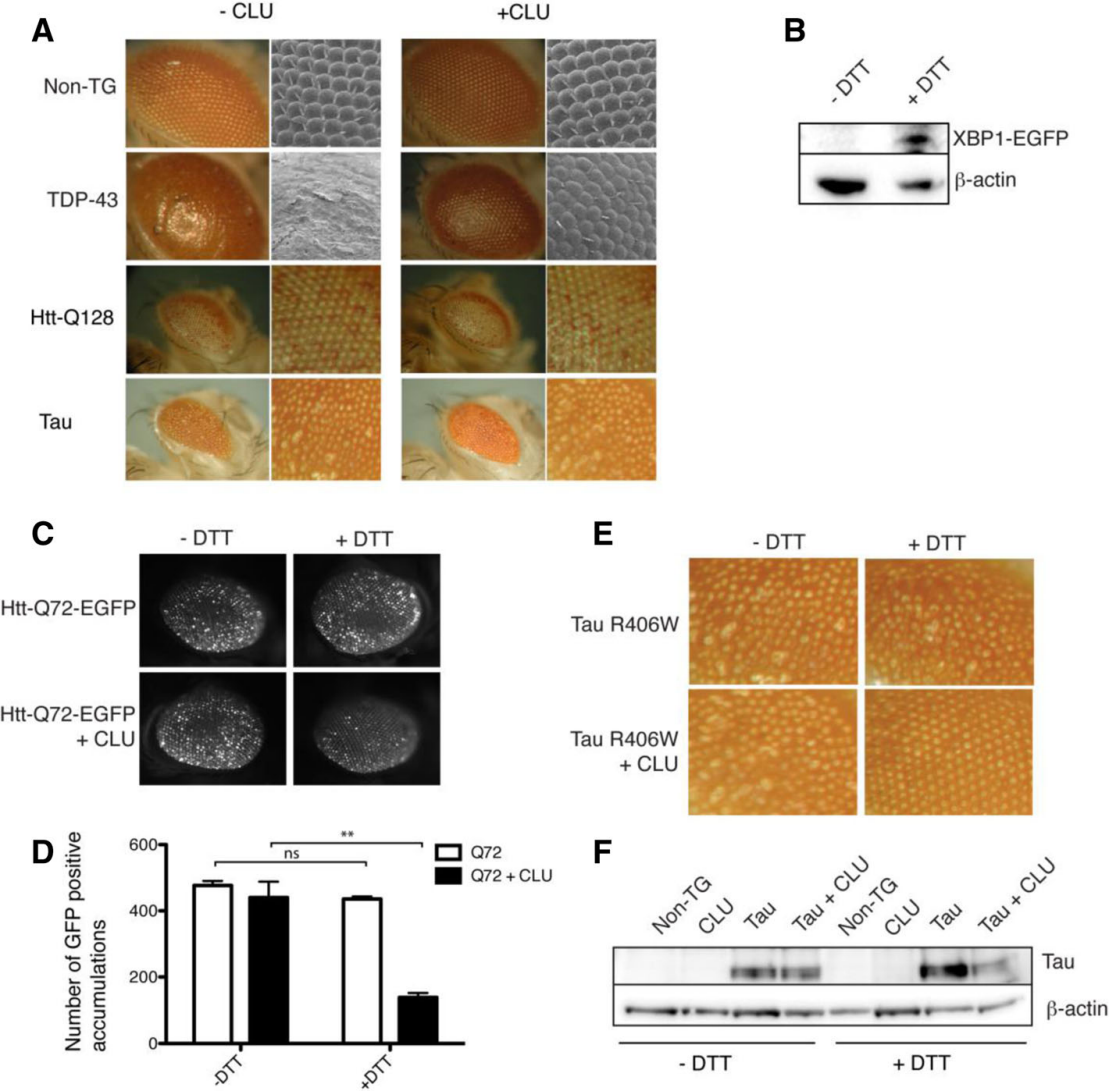
CLU provides ER stress-dependent protection against proteotoxicity. **a** Light and scanning electron micrographs demonstrating the effects of expression of TDP-43, Htt-Q128 and tau R406W (+/− CLU) in the photoreceptor neurons of adult *Drosophila*. Light micrographs (*left*) of *Drosophila* eyes collected using a 7X objective, electron micrographs (*right*) taken at 200X magnification. For Htt-Q128 and tau R406W, the images shown on the right are optical zooms of the corresponding images on the left. All images are representative of many experiments. **b** Western blot of whole non-transgenic *Drosophila* head lysates prepared from *Drosophila* fed normal food (−DTT) or food supplemented with DTT (+DTT); detection of XBP1-EGFP indicates activation of the UPR (β-actin was used as a loading control). **c** Fluorescence micrograph images (collected using a 7X objective) of eyes on *Drosophila* fed with food +/− DTT (or not), and expressing Htt-Q72-EGFP +/− CLU*.* **d** Quantification of the number of individual EGFP accumulations per eye, using images such as those shown in (**c**) and ImageJ (particle analyser program); **p = 0.0037, n = 9, Student’s t-test. Results shown are representative of several independent experiments. **e** Light micrographs (collected using a 7X objective) showing the eye phenotype in adult *Drosophila* fed normal food (−DTT) or food supplemented with DTT (+DTT) resulting from expression of R406W tau +/− CLU in the photoreceptor neurons. **f** Western blot of *Drosophila* head homogenates prepared from non-transgenic (Non-TG) *Drosophila* or *Drosophila* described in (**e**)

We used the “rough eye” assay as a widely accepted tool to assess neurotoxicity in *Drosophila* models, to test of the effects of CLU expression on a variety of proteotoxic stresses. The *gmr-GAL4* promoter was used to express TDP-43 in *Drosophila* photoreceptors, resulting in neurotoxicity manifested as a depigmentation and structural derrangement of the ommatidia, which was significantly reduced by CLU expression (Fig. 6a). We next expressed two other neurotoxic proteins (Huntingtin-Q128 (Htt-Q128) and mutant R406W human tau), which we had earlier established did not induce ER stress in *Drosophila* neurons (Fig. 3c). In both these cases, CLU co-expression had no significant effect (Fig. 6a). We reasoned that the lack of protection against proteotoxicity afforded by CLU in these models could relate to its known dependence upon ER stress for release from the ER to the cytosol. To examine this possibility we next expressed in the *Drosophila* eye the Htt gene (exon 1) with a 72 residue glutamine expansion, which can be readily visualized through its fused EGFP tag (Htt-Q72-EGFP) [43]. We then tested whether CLU co-expression could protect from the resulting aggregation and neurotoxicity during (i) basal conditions, and (ii) chemically-induced ER stress induced by rearing *Drosophila* on food supplemented with 5 mM DTT.

Western blot analysis of the XBP1-EGFP reporter in *Drosophila* head homogenates showed that rearing *Drosophila* on DTT-supplemented food is sufficient to induce ER stress, indicated by induction of the UPR (Fig. 6b). When comparing between *Drosophila* all co-expressing Htt-Q72-EGFP and CLU, relative to *Drosophila* fed on standard food, ER stressed *Drosophila* showed an approximately 70% reduction in the number of fluorescent Htt-Q72-EGFP puncta detected (440.4 ± 47.8 vs 138.4 ± 13.5; respectively; *p* = 0.0037, *n* = 9). This effect was absent when *Drosophila* expressing only Htt-Q72-EGFP were raised on food supplemented with DTT (Fig. 6c&d). Similarly, when *Drosophila* co-expressing R406W tau and CLU were reared on food supplemented with 5 mM DTT to induce ER stress (but not otherwise), there was a rescue of the rough eye phenotype (Fig. 6e) and a concomitant reduction in the quantity of phosphorylated, insoluble R406W tau deposits detected by SDS PAGE (Fig. 6f). These data indicate that CLU-mediated protection against the aggregation and neurotoxicity of Htt-Q72 and R406W tau is completely dependent upon ER stress (Fig. 6c-f).

## Discussion

Although the precise pathway of release remains to be determined, multiple studies now strongly suggest that ER stress triggers the release of post-translationally modified CLU from the ER to the cytoplasm [15–17] (see also Additional file 1: Figure S1). This may well explain the many previous reports that, under a variety of disease conditions, CLU occurs in cellular locations outside the secretory system, including the co-localization of CLU with intracellular tau tangles in Alzheimer’s disease [44], α-synuclein rich Lewy bodies in Parkinson’s disease [45], and inclusion bodies in myofibrillar myopathies [46]. It has also been reported that in response to chemotherapeutic drug treatment, CLU relocates to mitochondria and interacts with Bax to inhibit apoptosis [47]. There is abundant evidence from transgenic mouse models of ALS, neuropathological post mortem studies, and genetic studies that ER stress is a significant component in ALS pathology [48, 49]. While certainly not in themselves definitive, the results of our own immunohistochemical analyses of ALS patient spinal cord tissues are consistent with CLU also being released from the ER in ALS affected neurons (Additional file 1: Figure S2).

Furthermore, the inappropriate mislocalization and accumulation of TDP-43 in cytoplasmic inclusions is a unifying pathology in frontotemporal dementia (FTD) and familial and sporadic ALS cases. It is seen, with the exception of cases resulting from SOD1 mutations [50], in most post-mortem cases of FTD and ALS [51], and correlates well with synaptic pathology and cognitive deficits [52]. Thus, a therapeutic strategy that protects against TDP-43 proteotoxicity could provide benefits in most cases of ALS. Therefore, in the present study we used a variety of complementary approaches, including in vitro, cell and whole animal models, to evaluate whether the unusually potent and multifunctional chaperone CLU can affect the aggregation, cytoplasmic mislocalization and proteotoxicity of TDP-43.

We showed using both a C-terminal peptide corresponding to the most aggregation-prone region of TDP-43, and full length in vitro translated TDP-43-tGFP, that CLU potently inhibits the in vitro aggregation of TDP-43 and maintains it in a soluble form (Fig. 2). These results strongly suggest that CLU can directly interact with TDP-43, as there is no known mechanism by which a chaperone can inhibit protein aggregation without directly interacting with the client protein. The ability of CLU to inhibit the aggregation of the TDP-43^286-331^ peptide even at molar ratios of CLU:peptide as low as 1:1000 could arise from the fact that only a very small fraction of the soluble peptide molecules may be in a misfolded conformation at any one time, enabling CLU to interact specifically with these species to stabilize them and prevent them acting as aggregation nuclei [10]. We also showed using three different transfected cell models (N2a, SH-SY5Y and U251), that during ER stress, but not otherwise, CLU co-localized with cytoplasmic TDP-43-GFP inclusions (Fig. 1, Additional file 1: Figure S3). The treatments used to induce ER stress in the cell models (MG132, Tg and A23187) have been widely used in other studies [15–18] and we confirmed that even an 18 h exposure of N2a cells to 10 uM MG132 had negligible effects on cell viability (Additional file 1: Figure S4). In N2a cells, we showed that over-expression of CLU specifically and significantly reduced the numbers of inclusions within these cells (Fig. 2c). Co-immunoprecipitation analyses demonstrated that CLU can physically associate with TDP-43^CTF^-GFP in N2a cell lysates (Fig. 1b), suggesting that once in the cytosol, CLU would be capable of forming stable complexes with TDP-43.

In transgenic *Drosophila* we showed that overexpression of TDP-43 induced both ER stress and the retention of CLU within both photoreceptor and motor neurons (Fig. 3). Under these conditions, CLU was shown to co-localize with TDP-43 first in the cytoplasm and then, at later stages in development, in the nuclei of adult *Drosophila* motor neurons, effectively restoring the normal nuclear distribution of TDP-43 (Fig. 4). In this whole animal model, the effects of enforced CLU expression were to generate a dramatic recovery of the loss of locomotor activity associated with TDP-43 proteotoxicity, and a significant extension of lifespan (Fig. 5). These striking effects are specific to CLU expression as we showed that the co-expression of GFP in TDP-43-expressing *Drosophila* did not alter either the level of TDP-43 expression or the median survival (Additional file 1: Figure S7). Finally, we showed that enforced CLU expression could also protect against the proteotoxicity of Htt-Q72 and R406W tau expressed in *Drosophila* photoreceptor neurons, but only when ER stress was chemically induced (Fig. 6).

In both the neuronal cell and *Drosophila* models studied here, ER stress was a consistent requirement for CLU to provide protection against protein mislocalization and toxicity. We cannot exclude the suggestion that over-expression of CLU might act to reduce the levels of experimentally induced ER stress, and this might account in part for the protective effect of CLU expression. However, even if this were correct, our results in transgenic *Drosophila* strongly suggest that this is not the only mechanism by which CLU over-expression can protect against the cytoplasmic accumulation of toxic proteins. Notably, the level of cytoplasmic accumulation/toxicity of Htt-Q72/R406W tau expressed in cells of the *Drosophila* eye is unaffected by either DTT treatment alone or CLU co-expression alone, but is significantly reduced only when the two conditions are combined (Fig. 6). Furthermore, in transfected N2a cells, we only measure the release of CLU to the cytosol following treatments to induce ER stress, again strongly suggesting that CLU over-expression does not ablate the induction of ER stress. All our findings are consistent with a model in which ER stress induces release of CLU to the cytosol where it exerts effects that act to reduce net TDP-43 accumulation. There are a several putative mechanisms by which cytosolic CLU could exert the observed effects on TDP-43 mislocalization, aggregation, toxicity and clearance. These include (i) the well characterized chaperone activity of CLU [11, 12, 53] allowing it to directly interact with TDP-43 to form stable, soluble complexes, thereby inhibiting its further aggregation to form cytoplasmic inclusions, (ii) CLU may interact with misfolded species of TDP-43 to neutralize their toxicity, as is the case in its interactions with other toxic protein oligomers [12, 54, 55], and (iii) CLU, which is ubiquitinated once in the cytosol [15], may direct soluble CLU-TDP-43 complexes towards the proteasome or autophagy for degradation. Both the proteasome and autophagy are known to act to clear TDP-43 inclusions [56] and there is a growing body of evidence implicating CLU as a substrate of both these major cellular degradative pathways and a control element of autophagy [15, 19–22]. It is also interesting to note in this context that Cha-Molstad et al. have recently reported that in response to MG132-mediated inhibition of the proteasome, or cytosolic foreign DNA, the ER-resident chaperone BiP is released to the cytosol [18]. We speculate therefore that the release of CLU to the cytosol might form a part of a larger strategy in which multiple chaperones are released from the ER to the cytosol to defend the cell against intracellular stresses.

An important aspect of motor neurons is that they have a very limited heat shock response owing to their impaired ability to activate the key molecular heat shock response molecule, HSF1 [57]. This results in a high threshold for induction of the heat shock response in motor neurons conferring an element of vulnerability. The results presented in this study provide a clear demonstration in a whole organism model that during ALS disease-relevant conditions the enforced expression of CLU provides significant protection against the TDP-43-mediated proteotoxicity underpinning pathology, to substantially enhance motor neuron survival, reduce locomotor deficits and extend lifespan. We have shown previously that constitutively intracellular chaperones are able to rescue TDP-43 associated neurotoxicity [14], however this is the first instance, to our knowledge, where a normally secreted chaperone can exert similar protective effects.

In protein misfolding diseases such as ALS and Alzheimer’s disease (AD) there are multiple proteins misfolding in both the intra- and extracellular environments. For example, pathological, extracellular amyloid beta accumulation is often seen in post-mortem ALS and AD cases exhibiting intracellular accumulation of TDP-43 or tau [58]. It appears feasible that CLU can combat protein misfolding in both the intra- and extracellular compartments and may alter its subcellular localization dependent upon the environmental stress. Previous studies attempting to harness the therapeutic benefits of chaperones in protein misfolding diseases have used Hsp90 inhibitors, such as 17-AAG, which can upregulate small heat shock proteins in the intracellular compartment. 17-AAG can rescue the neurotoxicity associated with the pathological accumulation of TDP-43, polyglutamine, alpha synuclein, mutant androgen receptor, tau and amyloid beta [59–63]. However, 17-AAG also upregulates CLU expression [64], potentially contributing to the neuroprotective effects afforded by the therapeutic intervention in these models. Apart from ALS, many other neurodegenerative diseases including Alzheimer’s, Parkinson’s and Huntington’s diseases, also feature ER stress associated with pathology [23]. Therefore, it is conceivable that further studies investigating temporal changes in CLU levels and localization associated with pathologies could reveal new avenues for developing therapeutic strategies to address currently incurable and highly devastating diseases.

## Conclusions

The results presented in this study demonstrate in a whole organism model that during ALS disease-relevant conditions, enforced CLU expression significantly protects against TDP-43-mediated proteotoxicity to substantially enhance motor neuron survival, reduce locomotor deficits and extend lifespan. This is the first instance, to our knowledge, where a normally secreted chaperone has been shown to exert such protective effects in the intracellular context.

## Additional file

Additional file 1: Supplementary Methods. **Figure S1.** In mouse N2a neuroblastoma cells, endogenous CLU is released from the ER to the cytosol in response to treatment with MG132 and Tg. **Figure S2**. Spinal cord anterior horn cells of ALS patients contain phospho-TDP-43 inclusions and elevated levels of abnormally distributed intracellular CLU. **Figure S3.** ER stress-dependent co-localization of CLU with cytoplasmic TDP-43 inclusions in different transfected cell models. **Figure S4.** Treatment of N2a cells with 10 μM MG132 for 18 h does not cause significant loss of cell viability. **Figure S5.** (A) CLU reduces the number of TDP-43^M337V^-GFP inclusions in response to ER stress. Transfected N2a cells were treated as indicated and then lysed and fluorescent protein inclusions were enumerated by flow cytometry. (B) Raw flow cytometry data showing that expression of CLU or mCherry in unstressed (untreated) transfected N2a cells expressing TDP-43^M337V^-GFP has no significant effect on numbers of inclusions. **Figure S6.** Western blot data relating to the CLU *Drosophila* model. **Figure S7.** Overexpression of GFP does not suppress expression of TDP-43, and additional *Drosophila* survival data. (DOCX 1057 kb)
