# Supplementary material for: Clusterin protects neurons against intracellular proteotoxicity
Source: Acta Neuropathol Commun. 2017 Nov 7;5:81. doi: 10.1186/s40478-017-0481-1 (PMC5678579; doi:10.1186/s40478-017-0481-1)
Supplement: Additional file 1: — Supplementary Methods. Figure S1. In mouse N2a neuroblastoma cells, endogenous CLU is released from the ER to the cytosol in response to treatment with MG132 and Tg. Figure S2. Spinal cord anterior horn cells of ALS patients contain phospho-TDP-43 inclusions and elevated levels of abnormally distributed intracellular CLU. Figure S3. ER stress-dependent co-localization of CLU with cytoplasmic TDP-43 inclusions in different transfected cell models. Figure S4. Treatment of N2a cells with 10 μM MG132 for 18 h does not cause significant loss of cell viability. Figure S5. (A) CLU reduces the number of TDP-43M337V-GFP inclusions in response to ER stress. Transfected N2a cells were treated as indicated and then lysed and fluorescent protein inclusions were enumerated by flow cytometry. (B) Raw flow cytometry data showing that expression of CLU or mCherry in unstressed (untreated) transfected N2a cells expressing TDP-43M337V-GFP has no significant effect on numbers of inclusions. Figure S6. Western blot data relating to the CLU Drosophila model. Figure S7. Overexpression of GFP does not suppress expression of TDP-43, and additional Drosophila survival data. (DOCX 1057 kb) [file 40478_2017_481_MOESM1_ESM.docx]

**Additional file**

**Additional file 1: Supplementary Methods**

*Cell fractionation and Western blotting*

N2a cells were incubated without (control) or with 10 μM MG132 and 2.5 μM thapsigargin (Tg) for 20 h to inhibit the proteasome and induce ER stress. Cells (~ 5 x 10^6^ per treatment) were then processed using a Subcellular Protein Fractionation Kit (ThermoScientific) following the manufacturer’s instructions. The “cytosolic fraction” (CF) and the “membrane fraction” (MF; containing ER proteins) were concentrated 20-fold using ultrafiltration concentrators (Millipore; 30 kDa cut-off). The concentrated fractions were diluted 1:1 with non-reducing 2X SDS PAGE sample buffer and heated at 100 ^o^C for 5 min before electrophoresis on a 10% SDS PAGE gel and then overnight transfer (30 V at 4 ^o^C) to nitrocellulose membrane (BioTrace NT; Pall Life Sciences). The membranes were subsequently incubated at room temperature for 1 h in blocking buffer (5% w/v skim milk powder in PBS), and then sequentially incubated with primary and HRP-conjugated secondary antibodies diluted in blocking buffer. Primary antibodies used were monoclonal anti-mouse clusterin antibody (1:400, Sino Biologicals Inc.) and a mixture of monoclonal anti-BiP antibody (1:2500, Abcam) and monoclonal anti-β-tubulin antibody (1:2500, Sigma Aldrich). Goat anti-mouse IgG-HRP and goat anti-rabbit IgG-HRP (Dako, Ely, UK) were used at 1:500. Specific protein bands were detected using Supersignal Western Pico Substrate (Pierce) following the manufacturer’s instructions and chemiluminiscence imaged using an A600 Gel Imager (Amersham Biosciences).

*Human Spinal Cord Immunohistochemistry*

All post-mortem tissue was collected via the Edinburgh Brain Bank, approved by a national ethics committee, in line with the Human Tissue (Scotland) Act. Use of human tissue for post-mortem studies has been reviewed and approved by the Sudden Death Brain Bank ethics committee and the Academic and Clinical Central Office for Research and Development (ACCORD) medical research ethics committee (AMREC). Three ALS cases and three age- and sex-matched controls were used for these analyses. Spinal cord was taken at postmortem and fixed in 10% formalin for a minimum of 24 h. Tissue was dehydrated in an ascending alcohol series (70-100%) followed by three successive 4 h washes in xylene. Three successive 5 h paraffin wax embedding stages were performed followed by cooling and sectioning of the formalin-fixed paraffin-embedded tissue on a Leica microtome in 4 μm sections on to superfrost microscope slides. Sections were dried overnight at 40 °C and immunostaining was performed using the Novolink Polymer detection system with the Proteintech anti-phospho(409-410)-TDP-43 antibody at a 1 in 1000 dilution, G7 & 41D mouse-anti-CLU antibodies (undiluted cell hybridoma supernatant) and visualized using DAB chromogen, counterstained with hematoxylin, according to standard operating procedures. The slides were then cleared in xylene and mounted with a 24 x 50 mm coverslip using 2 drops of VectaMount mounting medium. Sections were imaged at 20x and 40x magnification on a NanoZoomer S60 digital slide scanner and representative images were selected using the NanoZoomer NDP.view2 viewing software.

**Additional file Figures and Figure Legends**

**Additional file 1: Figure S1.** In mouse N2a neuroblastoma cells, endogenous CLU is released from the ER to the cytosol in response to treatment with MG132 and Tg. A Subcellular Protein Fractionation Kit (Thermo Fisher Scientific) was used to prepare cytosol fractions (CF) and ER-enriched “membrane” fractions (MF) from N2a cells incubated for 20 h without (untreated) or with 10 uM MG132 and 2.5 uM Tg. The latter treatment will inhibit the proteasome and induce ER stress. These fractions were then analysed by immunoblotting. Validating the purity of the cell fractions obtained, left of the molecular weight markers, specific bands of the expected masses are detected for β-tubulin in the CF but not in the MF, and for BiP in the MF but not in the CF (multiple bands are routinely detected for BiP using this antibody; Abcam ab21685). To the right of the molecular weight markers, in untreated cells, CLU is detected in the MF but not in the CF. In contrast, in the treated cells, CLU is detected in both the CF and the MF confirming its release from the ER to the cytosol under these conditions. The apparent ~ 55 kDa of the CLU detected is consistent with its known limited glycosylated inside the ER ^1, 2^.

**
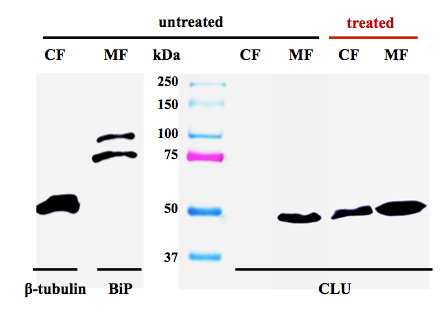
**1. Kapron, J.T. *et al.* Identification and characterization of glycosylation sites In human serum clusterin. *Protein Sci.* **6**, 2120-2133 (1997).

2. Collard, M.W. & Griswold, M.D. Biosynthesis and molecular cloning of sulfated glycoprotein 2 secreted by rat Sertoli cells. *Biochemistry* **26**, 3297-3303 (1987).

**Additional file 1: Figure S2. Spinal cord anterior horn cells of ALS patients contain phospho-TDP-43 inclusions and elevated levels of abnormally distributed intracellular CLU**

Bright field images of anterior horn cells of an ALS patient compared to an age- and sex-matched control. Images were collected using 20X (A) and 40X objectives (B). Sections were stained with anti-phospho-TDP-43 (pTDP-43, top panel) and anti-CLU (bottom panel) antibodies, and were counterstained with hematoxylin to visualize morphology. Scale bars are indicated. In both A and B, the images are representative of three patients and three age- and sex-matched controls. The blue arrow (upper left panel in B) indicates intracellular TDP-43 inclusions inside one anterior horn cell in the spinal cord of an ALS patient, not seen in control patient cells (also visible in A, upper panels). Lightly stained foci in control cells is Nissl substance, corresponding to the location of concentrations of rough endoplasmic reticulum (ER; white arrow, top right panel in B). The white arrow in the lower right panel of B shows specific detection of CLU in regions corresponding to Nissl substance, consistent with its primary location inside the ER. In ALS patient cells, relative to controls, the intensity of CLU staining is increased (A) and the pattern of CLU staining is more diffuse throughout the cell, not focused in the Nissl substance (red arrow, lower left panel in B). Staining with an isotype-matched control antibody (DNP9) was negligible. These results suggest that, relative to controls, human anterior horn cells containing pathological TDP-43 aggregates also contain elevated levels of CLU, which is abnormally localized throughout the cell.

**
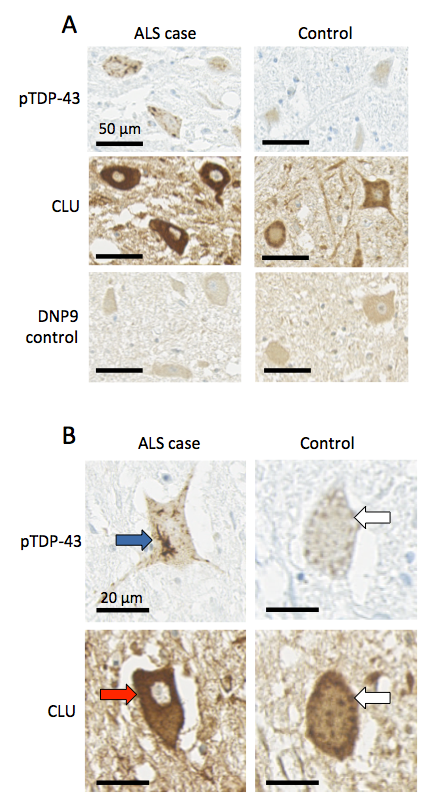
Additional file 1: Figure S2**

**Additional file 1: Figure S3.** ER stress-dependent co-localization of CLU with cytoplasmic TDP-43 inclusions in different transfected cell models. (A) SH-SY5Y cells transfected with TDP-43^CTF^-GFP show punctate cytoplasmic inclusion bodies both with and without Tg-induced ER stress. In unstressed cells, endogenous hCLU (detected using G7 monoclonal mouse antibody and Hilyte-647-conjugated secondary antibody) showed a distribution typical of its normal presence in the secretory system (ER, Golgi and secretory vesicles), but co-localized with TDP-43^CTF^-GFP inclusions during ER stress (visible as yellow, bottom right panel). (B) Similarly, transfected U251 cells expressing M337V TDP-43-tdTomato show punctate cytoplasmic inclusion bodies after being treated with MG132 and Tg. In this model, MG132 was necessary to induce inclusion formation in most cells. In unstressed cells, hCLU (detected using G7 monoclonal mouse antibody and Alexa Fluor488-conjugated secondary) showed the typical appearance of a distribution in the secretory system, but co-localized with TDP-43-tdTomato inclusions following MG132 + Tg treatment (visible as yellow, bottom right panel). DNA was stained with RedDot2. In both images the scale bar is 10 μm.

**
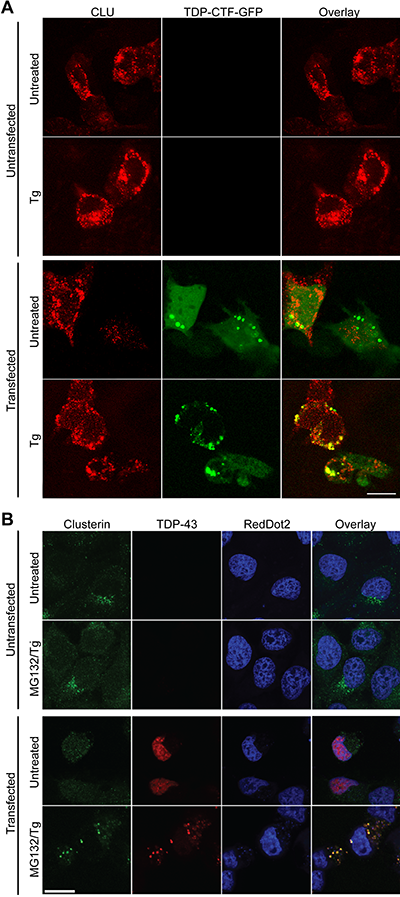
Additional file 1: Figure S3**

**Additional file 1: Figure S4.** Treatment of N2a cells with 10 μM MG132 for 18 h does not cause significant loss of cell viability. N2a cells were incubated in 96 well plates in 10% (v/v) fetal bovine serum in DMEM:F12 medium at 37 C in a CO_2_ incubator. Cells were supplemented with 0.1% (v/v) DMSO (treatment control) or MG132 at 10 μM or 100 μM (diluted from stocks in DMSO to give a final concentration of 0.1% DMSO) and incubated for 18 h before measuring cell death using propidium iodide (PI) fluorescence. Cells were stained for 20 min with 3 μM PI, gently washed with PBS, then 200 ul of PBS added to each well before reading fluorescence in a PolarStar microplate reader (BMG Labtech; excitation bandpass filter 544/10 nm, emission bandpass filter 620/10 nm). Values shown are means of 12 replicate wells and the error bars indicate standard deviations. Values have been corrected for buffer-only background fluorescence. Values for 0.1% DMSO and 10 μM MG132 are not significantly different (p > 0.05, Student’s t test). Flow cytometric analysis of cell viability confirmed that 18 h treatment with 100 mM MG132 induced ~ 80% loss of cell viability (not shown).


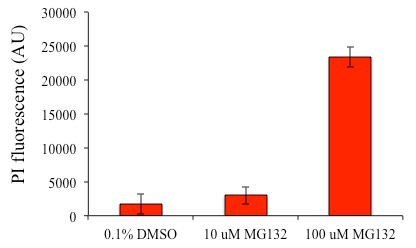


**Additional file 1: Figure S5** (A) CLU reduces the number of TDP-43^M337V^-GFP inclusions in response to ER stress. Transfected N2a cells were treated as indicated and then lysed and fluorescent protein inclusions were enumerated by flow cytometry. (B) Raw flow cytometry data showing that expression of CLU or mCherry in unstressed (untreated) transfected N2a cells expressing TDP-43^M337V^-GFP has no significant effect on numbers of inclusions. For both experiments, values are means + SEM, n = 3. Results are indicative of two independent experiments. ** p < 0.01, *** p < 0.001 (two-way ANOVA with a Bonferroni post-test).


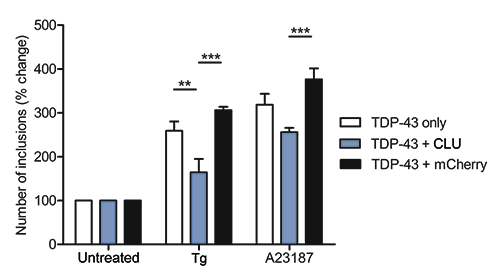


A


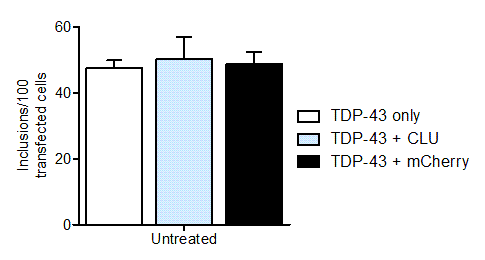


B

**Additional file 1: Figure S6.** Western blot data relating to the CLU *Drosophila* model. (A) Detection of the RIPA soluble fraction of CLU from non-reduced *Drosophila* head homogenates and the positive control (CLU purified from human plasma), showing that CLU is glycosylated to a lesser extent when expressed in *Drosophila* compared to humans. [+ DG]: deglycosylated for 6 h with PNGase, [- DG]: not deglycosylated. Individual panels are from the same Western blot, reordered for clarity. (B) Detection of human plasma CLU (left, reducing conditions) and CLU in haemolymph (right, non-reducing and reducing conditions). Black arrow indicates fully reduced CLU, red arrow indicates non-reduced CLU. Minor band at about 50 kDa in non-reduced hemolymph may represent a glyco-variant. (C) Detection of phosphorylated eIF2α, a marker for activation of the UPR, in *Drosophila* expressing TDP-43, compared to non-TG (non-TG) *Drosophila*. β-actin was used as a loading control. Results shown are each representative of several independent experiments*.*

**
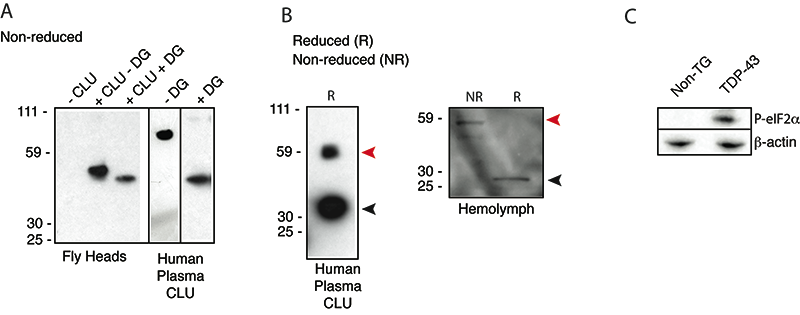
**

**Additional file 1: Figure S7.** Overexpression of GFP does not suppress expression of TDP-43, and additional *Drosophila* survival data. (A) Western blot detecting TDP-43 in adult *Drosophila* head homogenate**,** with and without co-expression of a control protein, GFP. The result indicates no detectable difference in TDP-43 levels when two proteins are co-expressed in *Drosophila*. (B) Survival of *Drosophila* expressing TDP-43 alone, TDP-43 with CLU and TDP-43 with UAS-GFP in adult motor neurons compared to heat shocked Non-TG *Drosophila* and *Drosophila* expressing only CLU and only UAS-GFP. Lifespans were analysed by Kaplan Meier statistics and show no significant difference in lifespan when TDP-43 is co-expressed with UAS-GFP compared to *Drosophila* only expressing TDP-43. (C) Median survival of *Drosophila* expressing TDP-43 and co-expressing (or not) human CLU, in adult motor neurons (*left*), compared to heat shocked non-transgenic (Non-TG) *Drosophila* with and without CLU expression (*right*). Lifespans were analysed by Kaplan Meier statistics. Non-significant (ns; p=0.5056) and significant (***; p=0.0006) results are indicated on the graph.

**
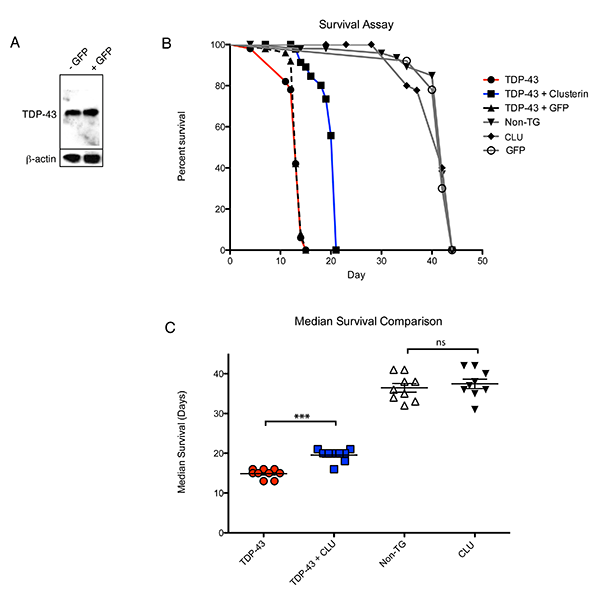
**
